# Non-toxic total nitrogen determination using a low alkaline persulfate digestion

**DOI:** 10.1016/j.mex.2020.100791

**Published:** 2020-01-16

**Authors:** Jacob L. Studt, Ellen R. Campbell, Dianna Westrick, Troy Kinnunen-Skidmore, Aimee H. Marceau, Wilbur H. Campbell

**Affiliations:** NECi Superior Enzymes, 334 Hecla Street, Lake Linden, MI 49945, USA

**Keywords:** Total nitrogen determination by alkaline persulfate digestion, Simple, Non-toxic, Nitrogen, Measurement, Water, Soil, Waste water, Agriculture

## Abstract

Measuring total nitrogen, nitrate, and nitrite is critical for compliance with water safety standards. Previous methods for measuring total nitrogen were hazardous, time consuming, and expensive. Here we report a method for measuring total nitrogen in water and soil using alkaline persulfate digestion combined with a Nitrate Reductase assay. In this method the alkaline persulfate reaction oxidizes all nitrogen present in the sample to nitrate, Nitrate Reductase then is used to catalyze the reduction of nitrate to nitrite in the presence of NADH. The nitrite is then treated with Griess reagents to produce a pink color. The absorbance of this color is measured at 540 nm using a spectrophotometer and when compared to a standard curve of nitrate, treated with both the reduction and colorizing steps, can be used to determine the total nitrogen content of measured samples. This method customizes the measurement of total nitrogen by combining alkaline persulfate digestion with a Nitrate Reductase assay using enzyme based green chemistry.

• Customization of total nitrogen analysis by combining alkaline persulfate digestion, driving all nitrogen to nitrate, with a colorimetric nitrate reductase assay
• Nitrate reductase catalyzes all nitrate, produced by alkaline persulfate digestion and present in the original sample, to nitrite
• Nitrite is measured by the addition of sulfanilamide and N-(1-napthyl)ethylenediamine dihydrochloride, resulting in a pink color

## Graphical abstract

**Figure:**
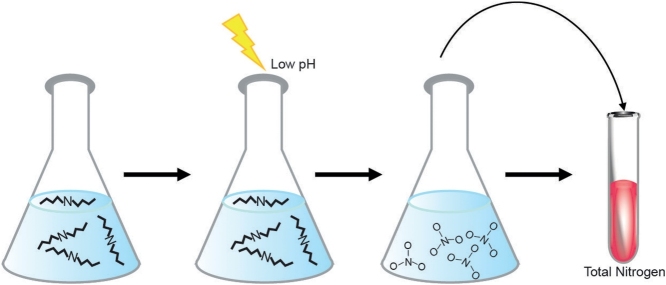

**Specification Table**

**Table:** 

Subject Area:	Environmental Science
More specific subject area:	Water and Soil Analysis of Total Nitrogen
Method name:	Total Nitrogen Determination by Alkaline Persulfate Digestion
Name and reference of original method:	J.J. Ameel, R.P. Axler, C.J. Owen, Persulfate digestion for determination of total nitrogen and phosphorus in low-nutrient waters, American Environmental Laboratory. 1 (1993) 10–11.
Resource availability:	https://nitrate.com/; https://instantpot.com/

## Method details

### Alkaline persulfate digestion

The alkaline persulfate digestion is used to completely oxidize nitrogenous compounds to nitrate for the determination of total nitrogen [1]. The quantitative analysis of total nitrogen has previously only been done with the cumbersome, hazardous Kjeldahl method [2,3] or alkaline persulfate digestion combined with toxic cadmium reduction of nitrate [3]. There are two additional methods [4,5] that utilize ion chromatography or utilize a proprietary instrument for high temperature catalytic oxidation and total organic carbon analyzer to assess total nitrogen. We present an environmentally-friendly, nontoxic alternative in which we use alkaline persulfate digestion combined with a Nitrate Reductase enzyme method for total nitrogen analysis.

Most persulfate digestion reactions initially start alkaline [3], although some methods suggest acidic yields a more complete digestion [6]. Independent of the method chosen, in the end of the digestion and thermal decomposition of persulfate, the pH will become highly acidic (pH 1–2) due to the high concentration of bisulfate ion generated from persulfate decomposition [see Eq. (1)]. The final pH is not important in regards to nitrogen oxidation, most compounds will be oxidized under alkaline conditions, but if there is an interest in phosphate, a final low pH is critical for the acid catalyzed hydrolysis and oxidation of phosphorus to orthophosphate.

(1) $${\text{S}}_{2}{\text{O}}_{8}^{2-}+{\text{H}}_{2}\text{O }\overset{\Delta }{\to }2\text{HS}{\text{O}}_{4}^{-}+1/2{\text{O}}_{2}$$

Samples containing chloride (or bromide or iodide, such as seawater) can be problematic, since chloride oxidizes to form chlorate ions [7]. Chlorate is an inhibitor and alternate substrate of nitrate reductase [8]. To prevent the formation of chlorate, an alkaline pH is required and can be accomplished by introducing an excess of hydroxide ions in the reaction [see Eq. (2)].

(2) $$3{\text{S}}_{2}{\text{O}}_{8}^{2-}+\text{C}{\text{l}}^{-}+6\text{O}{\text{H}}^{-}\to 6\text{S}{\text{O}}_{4}^{-2}+\text{Cl}{\text{O}}_{3}^{-}+3{\text{H}}_{2}\text{O}$$

A persulfate to hydroxide ratio of 1:2 must be present in the digesting solution, this will maintain a highly alkaline pH. The concentration of the digesting solution is not important but maintaining the ratio is required. The decomposition of persulfate is dependent on thermal degradation and not pH so nitrogen oxidation can occur in the presence of chloride without chlorate interference downstream.

The alkaline persulfate reaction oxidizes all organic nitrogen and ammonia in the sample to nitrate. This is a well known and accepted step in the determination of total nitrogen [3]. The next step in the current most common method is reduction of nitrate to nitrite by passing the sample over a granular copper-cadmium column. Cadmium is a known carcinogen. The method described here reduces the nitrate, produced by the digestion step, to nitrite using Nitrate Reductase and NADH. The Nitrate Reductase method is an EPA accepted method for measuring nitrate and nitrate in water [9]. In this method we have customized total nitrogen determination of environmental samples by combining alkaline persulfate digestion with an enzyme-based Nitrate Reductase assay.

## Methods

### Sample preparation

Collect forage samples and dry in an oven at approximately 60 °C for at least 24 h, or until the mass no longer changes. Dried forage samples are weighed to 1.0 g and placed in a 25 mL Erlenmeyer flask (Kimble™ KIMAX™ Narrow Mouth Erlenmeyer Flasks, or equivalent) along with 20 mL distilled water. Boil samples for a total of 20 min ensuring no samples boil dry. Recover extracts using a garlic press to remove as much extract as possible and store in labeled 15 mL Falcon™ tube (15 mL BD Falcon™ Centrifuge Tubes, polypropylene, or equivalent). Centrifuge tubes at 5k x *g* for 10 min then collect supernatant into a 10 mL volumetric flask, leaving pellet behind. Bring volumes to 10.0 mL with distilled water, concentrations are now 0.1 g/mL. Dilute samples appropriately (10, 100, 1000-fold), using a volumetric flask, for digestion reaction. After 1.0 mL of digestion solution is added, the concentration will become 0.083 g/mL which is important for calculating PPM N/g forage sample. For water samples take 5 mL of sample, the working range for the standard nitrate reductase assay is 0.5–10 Nitrogen mg/L, and mix with 1 mL of digestion solution. Dilute water samples with HPLC grade water to bring into the range of the standard assay.

### Alkaline persulfate digestion reaction

**Fig. 1:**
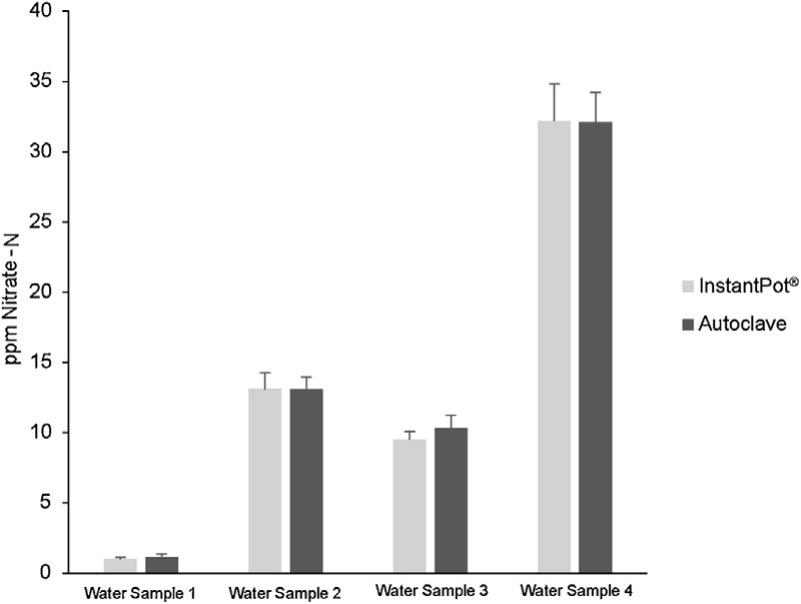
Comparison of total nitrogen analysis by alkaline persulfate digestion and nitrate reductase. 3 independent replicates of each water sample digested in either the Instant Pot® (light grey) or autoclave (dark grey) were averaged and plotted by ppm Nitrate – N.

The alkaline digesting solution was prepared by dissolving 1.31 g sodium persulfate (0.22 M Na_2_S_2_O_8_; Sigma, Catalog #S6172) in 50 mL 0.5 M sodium hydroxide (NaOH; Fisher Scientific, Catalog# S318), prepared fresh day of use. All water used was HPLC grade (Spectra; Catalog #HP902), and all reagents used were ACS Reagent grade or better. For total nitrogen samples, 5 mL sample of diluted extract, 5 mL of water sample, or 5 mL of nitrate standard was added to a Hach test tubes (Test ‘N Tube™ reactor/cuvette tubes, Catalog #25831, or equivalent), along with 1.0 mL alkaline digesting solution and mixed by vortexing. The volume of sample is not critical but the sample to digesting solution ratio of 5:1 must be maintained. For determining amount of nitrate in sample simply add 1.0 mL HPLC grade water in place of the alkaline digesting solution. Tubes are loosely capped then autoclaved at 121 °C, 15 psi, for 60 min. Allow tubes to cool to room temperature, assay for total nitrogen the day of digestion. The Instant Pot® serves as a cheap alternative to the autoclave. If using the Instant Pot® add 1 L of deionized water (not HPLC grade) in pot, place tubes in autoclave safe rack in pot. Attach lid and ensure steam release valve and handle are in closed position. Plug in pot and turn on pressure cook setting to high pressure, turn mode indicator to “More” and set timer for 1 h. Be sure to turn off the keep warm setting. After the run allow the cooker to cool down, wait until float valve has dropped. The entire cycle takes between 1.5 and 2 h. Samples were then quantitatively measured using Nitrate Reductase and the colorimetric Griess reagent [10,11]. Our work shows there is no significant difference in nitrate concentration between samples processed using the autoclave and those processed using the Instant Pot® (Fig. 1).

### Preparation of nitrate standards

**Fig. 2:**
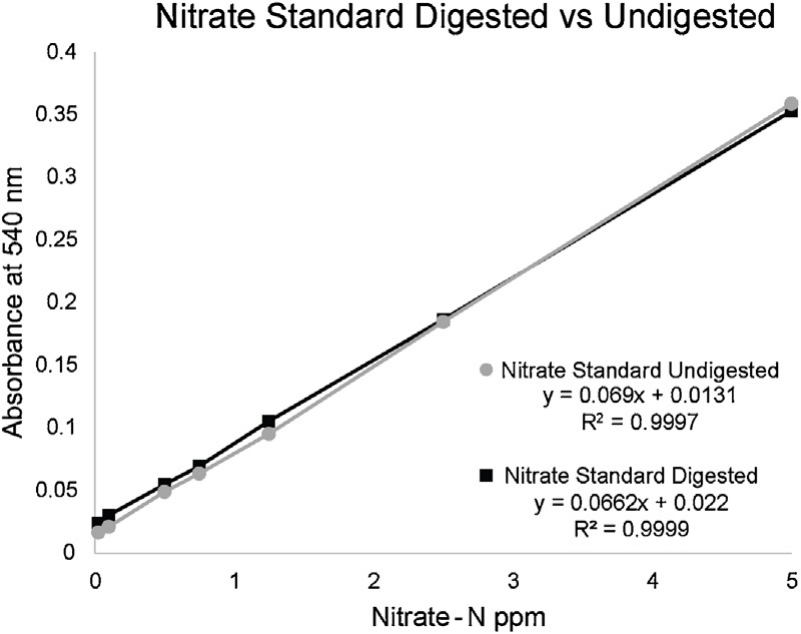
Preparation of nitrate standard. Nitrate standards were prepared and either digested by the alkaline persulfate method or left undigested. There is little to no difference between the nitrate standard curves.

Nitrate-N 1000 ppm standards were prepared by dissolving 1.44 g potassium nitrate (KNO3; Research Organics, Catalog No. 12011), in 200 mL HPLC water. Standards were then diluted to 10 ppm using a volumetric flask, prepared daily, and then diluted again to produce a standard range of 0.05–5.00 ppm for 7 data points. The standards are digested with alkaline persulfate or left undigested. Both sets of samples (digested and undigested) are tested with the nitrate reductase assay to determine the standard curve and show that the alkaline persulfate digestion does not impede nitrate recovery and measurement (Fig. 2). The standard curve is linear with a linear regression of 0.999. Digested versus undigested samples were prepared and data shows little difference (Fig. 2). Digested samples contain 1.0 mL digesting solution while undigested samples contained 1.0 mL distilled water. The comparison of the digested and undigested samples for the nitrate standard curve show that there is no loss of nitrate when performing alkaline persulfate digestion.

### Ammonium chloride - acidic persulfate versus low alkaline persulfate digestion

**Fig. 3:**
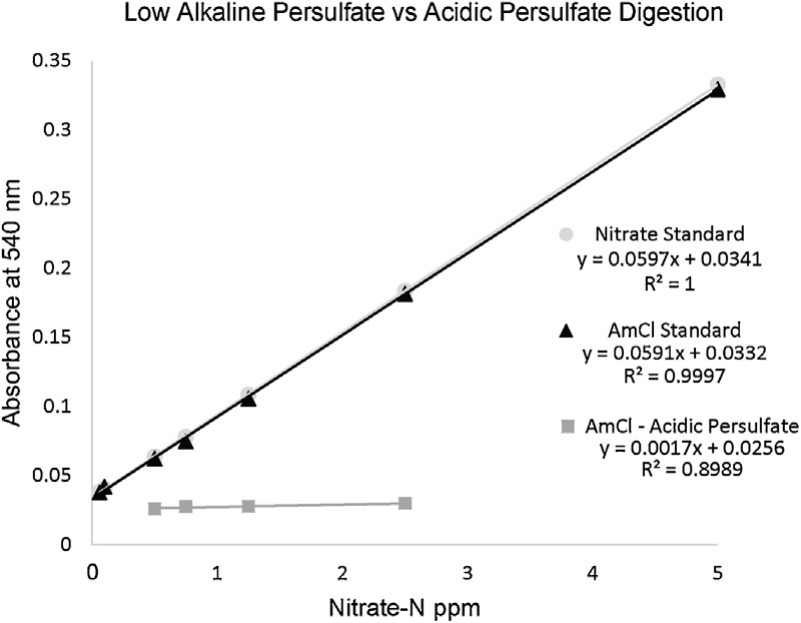
Comparison of Nitrate standard with Ammonium Chloride. Nitrate standard (grey circle) and ammonium chloride (AmCl) standards give very similar trend lines after low alkaline persulfate digestion. Under acidic persulfate digestion conditions the ammonium chloride standard (grey squares) produced chlorate which interferes with the nitrate reductase assay.

To determine if chlorate is being formed during the digestion, ammonium chloride-N 1000 ppm standards were prepared by dissolving 0.764 g ammonium chloride (NH_4_Cl; Sigma-Aldrich, Catalog #09718) in 200 mL HPLC water. To test for chlorate formation based on pH, two different methods were used. One experiment used the acidic persulfate method where the pH started highly acidic and remained highly acidic throughout the digestion. The other experiment used the low alkaline persulfate digestion where the pH starts highly basic and remains highly basic. The acidic persulfate digested ammonium chloride clearly shows inhibition with increased concentrations. Subsequently, the low alkaline persulfate method shows adequate nitrate-N recovery and the slope is agreeable with the nitrate standards (Fig. 3).

### Nitrate reductase assay - standard range - 0.5–5.0 ppm

Add 50 μL of digested or undigested sample to a labeled glass nitrate-free test tube (Borosilicate Glass 13 × 100 mm, Fisherbrand, Catalog #14-961-27, or equivalent). Add 900 μL assay buffer (0.1 M potassium phosphate containing 1 mM ethylenediaminetetraacetic acid (EDTA), pH 7.5) and 50 μL 2 mM nicotinamide adenine dinucleotide (NADH) to the sample tubes and vortex. Add 40 μL (0.5 U/mL) recombinant yeast NAD(P)H nitrate reductase (AtNaR, or YNaR1, EC 1.7.1.2) (1 unit = 1.0 μmole of nitrate to nitrite per minute, see Nitrate.com; or equivalent), vortex, and let tubes sit for one hour at room temperature. Add 500 μL color reagent #1 (1 % Sulfanilamide in 3 N HCl) and vortex. Add 500 μL color reagent #2 (0.02 % *N*-Naphthylethylenediamine in d-I water) and vortex then allow tubes to sit for 10 min. The contents of the reaction tubes are transferred to a polystyrene (PS) cuvette (Visible-Cuvette Disposable, Perfector Scientific, Catalog #9003, or equivalent) and the absorbance is read at 540 nm ± 20 nm in colorimeter or spectrophotometer for the samples and Nitrate Standards. To ensure accurate results, read absorbance between 10 and 30 min after color reagents are added. The absorbance at 540 nm for digested sample is compared to a digested standard curve prepared with certified KNO_3_ standard 1000 ppm diluted in distilled water to the range of 0.5–5.0 ppm. Using the linear regression equation of the standard curve, the total nitrogen content of each forage sample or water sample is calculated and recorded. If the results are outside the range of the nitrate standards simply dilute the digested sample appropriately and re-run the nitrate reductase assay.

## Additional information

Total nitrogen determination by alkaline persulfate digestion has several advantages over the traditional Kjeldahl method, these advantages have been described in previous literature [1,3] and we will discuss them here. Briefly the Kjeldahl method requires a strong acid and toxic metals for digestion of nitrogen containing compounds which creates issues with waste disposal, it requires large amounts of the samples to be tested (at least 50 mL), it requires special equipment. For the Kjeldhal method the accuracy is affected by ambient ammonia levels, uneven heating of the samples can give variable results, and the method is unsuitable for both high nitrate/nitrite levels and detection below 0.1 ppm. In contrast the alkaline persulfate method works with small sample volumes, there is no issue of ammonia contamination because all nitrogen containing compounds are oxidized to nitrate, and the equipment necessary is easily available. The customization described in this method shows that alkaline persulfate digestion works in both an Instant Pot® and autoclave. The United States Geological Survey (USGS) states that alkaline persulfate digestion is less toxic, more accurate and sensitive alternative to Kjeldahl digestion for nitrogen determination in environmental samples [3]. The USGS study showed that alkaline persulfate digestion results more that 95 % recovery of nitrogen containing compounds, the method of detection limit is 0.015 mg-N/L for total nitrogen compared to 0.05 mg-N/L for total Kjeldahl nitrogen [3]. In the USGS study they paired alkaline persulfate digestion with cadmium reduction, our method customization pairs alkaline persulfate digestion with an enzyme-based Nitrate Reductase assay. In the cadmium method the digested sample is passed through a column containing granular copper-cadmium which reduces the nitrate, created by the persulfate digestion, to nitrite which is then detected by use of color reagents. The limit of detection for this method is 0.05 mg/L nitrate-nitrite N. The primary issue with this method is the use of toxic cadmium and the requirement for specialized equipment. The nitrate reductase method described in this article works by a similar principle, the nitrate produced by the alkaline persulfate digestion is reduced to nitrite by the activity of the enzyme and detected by the use of color regents. The limit of detection for the Nitrate Reductase assay is 0.5 mg/L for the assay described here but can be as low as 0.05 mg/L with a few minor modifications. The Nitrate Reductase method described in this paper has been approved by the Environmental Protection Agency as an alternative to cadmium reduction of nitrate to nitrite [9]. The USGS has approved and published methods using Nitrate Reductase for the determination of nitrate and nitrite in water [12] (USGS Methods I-2547-11 and I-2547-11).
